# Germline organoids develop in vitro from embryonic *Taeniopygia guttata* (zebra finch) cultures

**DOI:** 10.1038/s41598-026-46600-z

**Published:** 2026-04-20

**Authors:** Bianca Brown, Elizabeth Nagy, Ajuni K. Takkar, Naomi A. Greengold, Shreyas S. Gujar, Ali Amini, Mary A. Collins, Javad Rajabi, Jenna Walls, Chenai Kaminski, Michael Sanchez, Elle McQuire-Guzman, Taraji Ellington, John R. Bracht

**Affiliations:** 1 Biology Department, American University, 4400 Massachusetts Avenue, NW, Washington, DC 20016 USA; 2 Center for Data Science, American University, 4400 Massachusetts Avenue NW, Washington, DC 20016 USA; 3 School of Public Affairs, American University, 4400 Massachusetts Avenue NW, Washington, DC USA; 4 Department of Computer Science, American University, 4400 Massachusetts Avenue, NW, Washington, DC 20016 USA

**Keywords:** Cell biology, Chromosomes

## Abstract

Organoids are three-dimensional structures that develop in cultures of stem cells, and resemble multicellular organs. While performing long-term culture of *Taeniopygia guttata* (zebra finch) germline tissue, we observed the formation of germline organoids from cultures of both sexes. These macro-scale structures contain multiple cell types and retain Primordial Germ Cells (PGCs) and their descendant germ-cell lineages for two to four months of culture *in vitro*. We show that the PGC-specific germline-restricted chromosome (GRC) can be detected after three months of culture, and that the organoids exhibit DAZL- and DDX4-positive structures resembling germinal epithelia. Taken together our results open new possibilities for the study of key steps of avian reproductive development.

## Introduction

Organoids are three-dimensional multicellular structures that capture some aspects of their source tissue and offer important biological insights^1^. For the germline, this includes the development of germ-cell lineages, which produce the gametes—the sperm and oocytes—along with somatic germline cells^2^. All gametes derive from Primordial Germ Cells (PGCs), and somatic germ-cell lineages provide supporting or steroidogenic roles^2^. In both birds and mammals, the PGCs are specified very early, before or during gastrulation^3^, and must find their way to the developing gonads. In avians, this journey is dramatic as PGCs circulate through the bloodstream before moving to the genital ridge, where they colonize and contribute to gamete formation^4^. In both males and females, the gonadal PGCs undergo first a proliferation stage (leading to a nest, or cyst) prior to the induction of meiosis^5,6^. In females, it is thought that each nest or cyst ultimately produces a follicle enclosing a single PGC-derived oocyte^7^, while in males the germline development produces seminiferous tubules; here, PGCs differentiate into spermatogonia, spermatocytes, and ultimately, sperm^8^.

The process of gonadal development including formation of gonadal structures, PGC homing, and PGC nest formation and breakdown, is generally opaque to observation *in vivo*. Birds compound this challenge by encasing their embryos within an opaque shell. Adding to this complexity, songbirds have also been documented to carry extra genetic material, the Germline-Restricted Chromosome (GRC), only in germ cells^9^, which would mean it is restricted to PGCs (and their descendant germ-cell lineages). In order to study the GRC, we adopted the PGC culture method previously published^10^ which begins with dissection of germline embryonic zebra finch tissue. However, while the previous study^10^ involved pooling of PGCs from multiple embryos, we cultured PGCs from single embryos on their own. Under these conditions we observed robust and regular organoid formation without any special 3D scaffolding and using standard 24-well cell culture plates. We observe that the organoids cluster with apparent PGCs *in vivo*. We show that organoids have a complex chambered internal structures, and that germ cells and gonadal somatic cells can be identified in epithelial structures and also sometimes within the organoids. We document the rapid growth and macro-scale of the organoids, which can grow within 21 days after dissection to sizes visible to the unaided eye (approx. $1 mm^2$). The behavior of these organoids is surprisingly dynamic, as they sometimes fuse with other organoids and some display an opening and closing behavior that can be measured in a few hours. These organoids open up research avenues for biologically vital germline developmental processes that are challenging to interrogate *in vivo*.

## Results

**Fig. 1:**
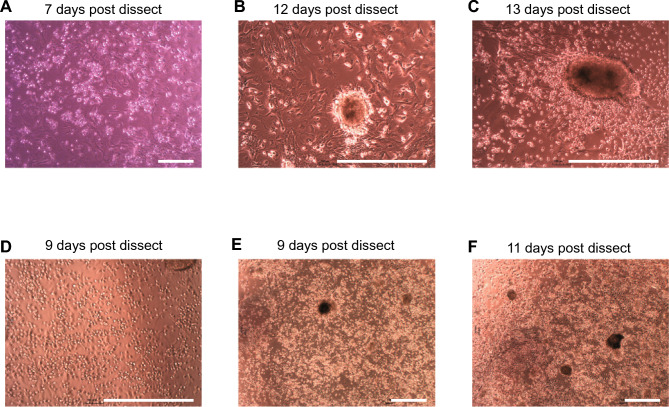
Early stages of organoid formation post dissection. (**A**) Seven days post dissection image from female; (**B**) 12 days post dissection image from female showing newly formed organoid; (**C**) 13 days post dissection image from the same well as (**B**) showing clustering of PGC’s around the organoid; (**D**) Nine days post dissection image from male; (**E**) Nine days post dissection image from male from a different well than (**D**) showing formation of organoids; **F**: 11 days post dissection image from the same well as (**E**) showing formation of more organoids. *Note* (**A**) and (**D**) are from different wells than their counterparts (**B**,** C**) and (**E**,** F**) respectively. Scale bar: 500 µm.

Following previous methods we established PGC culture *in vitro* from dissected zebra finch *Taeniopygia guttata* embryonic gonads^10^. However, we observed a distinct clustering of PGCs in many of our cultures and that within two weeks structures began forming in cultures from both sexes (Fig. 1A–F) In the female sample, we observed newly formed organoids by day 12 (Fig. 1B), apparently with PGCs clustering around the organoid more prominently by Day 13 (Fig. 1C). Male samples showed organoid formation as early as 9 days post-dissection (Fig. 1E), with multiple small organoids forming by day 11 (Fig. 1F).

**Fig. 2:**
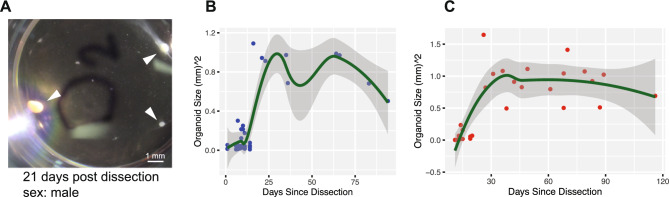
(**A**) 21 days post dissection organoids visible to the naked eye, scale bar: 1 mm. (**B**) Graphical depiction of size of male organoids over a span of 94 days. (Size data from n=36 total measurements, obtained from n=6 distinct organoids from days 0 to 30, n=3 organoids from days 31-70, and n=2 organoids from day 71 to the end. In total n=7 distinct male organoids were measured.) (**C**) Graphical depiction of size of female organoids over a span of 116 days. (Size data from n=30 total measurements, obtained from n=6 distinct organoids from days 0 to 30, n=4 organoids from days 31-70, and n=2 organoids from day 71 to the end. In total, n=9 distinct female organoids were measured.) To generate plots shown in (**B**) and (**C**) some of the same organoids were measured on different days of growth. For both plots, the dark green line is a best-fit local polynomial regression and the grey shading represents the 95% confidence interval.

**Fig. 3:**
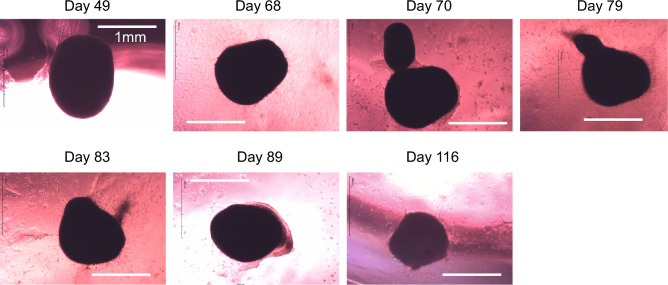
Stability in the size of mature organoid from 49 to 116 days post-dissection. Day 70, 79, and 83 show merging with a smaller organoid. Scale bar: 1000 µm.

**Fig. 4:**
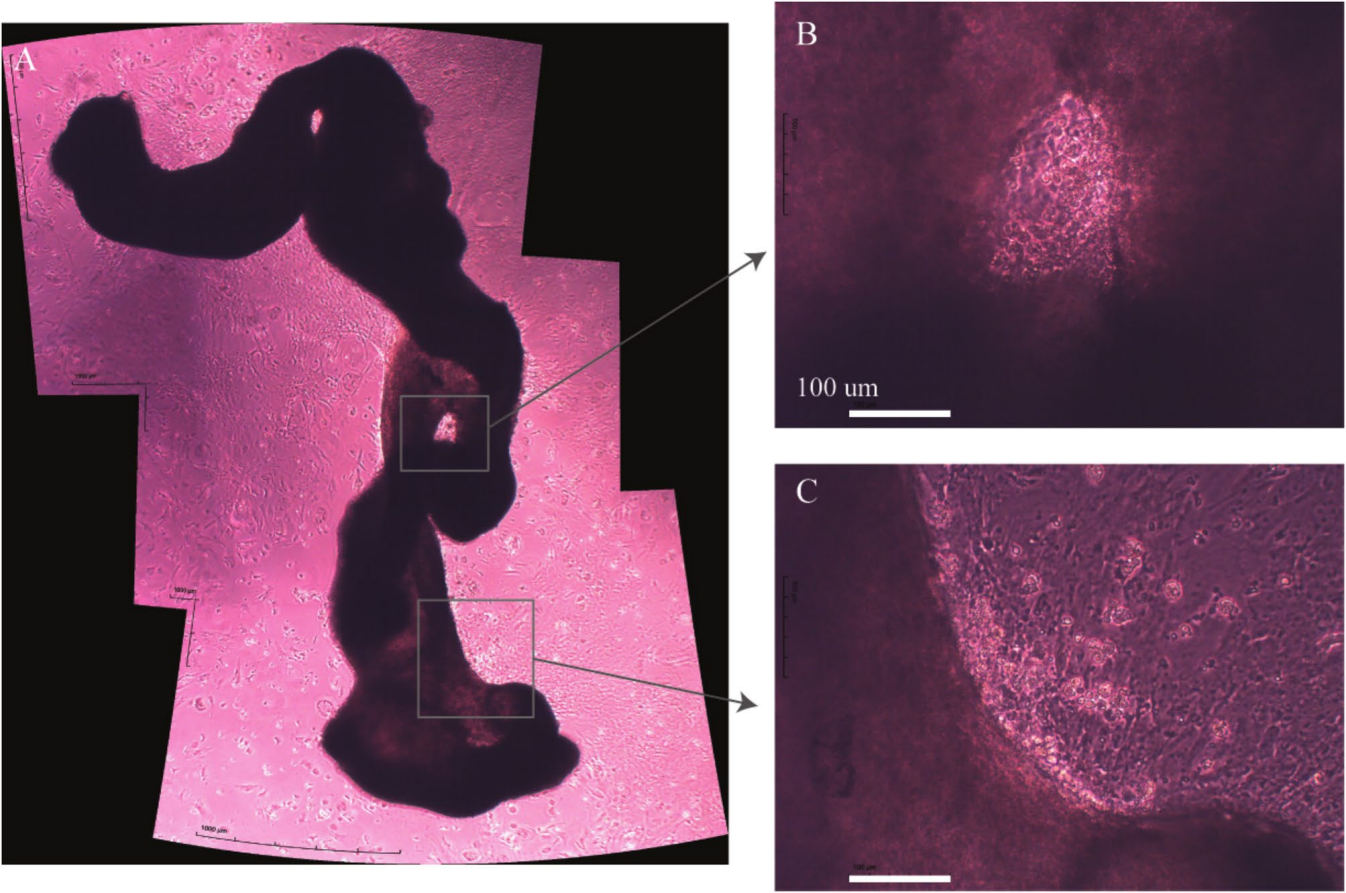
(**A**) Formation of a new organoid and clustering of PGCs; (**B**) Focusing on compartment visible inside the organoid showing Primordial Germ Cells (PGCs); (**C**): Focusing on the edge of the organoid showing region of dense PGCs. Scale bar: A: 1000 µm, B and C: 100 µm.

The organoids continued to grow over time, becoming visible to the naked eye (often reaching 1 mm across) by 21 days post-dissection (Fig. 2A). Using ImageJ software we tracked the size of multiple organoids for around 120 days for female organoids and 90 days for male organoids (Fig. 2B,C). The growth pattern suggests that organoids reach a stable size (approx. $1mm^2$) after an initial growth phase lasting 25 to 30 days (Fig. 3). We were able to document the stability in size of a single organoid from 49 to 116 days post-dissection (Fig. 3). Interestingly, a smaller organoid merged with this larger organoid on days 70, 79 and 83, but the structure maintained its overall size and stability (Fig. 3). When combined with data showing a general stasis of the organoid sizes over months of culture (Fig. 2B,C), a possible explanation is homeostatic size regulation, although we cannot rule out growth limitation due to nutrient diffusion.

Organoids generally end up as rounded structures, but we observed one rapidly forming organoid of highly irregular structure (Fig. 4A). By the following day the organoid had become a spherical structure. In this nascent organoid (Fig. 4A), we could see a clustering of what appear to be PGCs surrounding its borders: Fig. 4B focuses on a central region, while Fig. 4C highlights a region of what appear to be PGCs at the edge of the forming organoid. This raised the question of whether PGCs might be found within the organoids.

To test for germline cell lineages within the organoids we performed immunofluorescence on fixed organoid sections using antibodies for five germline-associated or PGC marker genes: DAZL, DDX4, EMA-1, SOX-9, and Aromatase (Table 1). Three of these (DAZL, DDX4, and SOX-9) are new to zebra finch. To maximize our chance of finding functional cross-reacting antibodies we performed a three-stage evaluation. First we identified polyclonal antibodies, which target multiple epitopes, making it more likely they would cross react. Second, we performed BLASTp analysis of the antigen relative to zebra finch, given that a 60% or better sequence identity is associated with increased probability of cross-reaction^11^. For the chosen antibodies, antigen sequences were 60% (DAZL), 67% (DDX4) and 97% (SOX-9) identical to zebra finch (Table 3). Third, we performed immunofluorescence of zebra finch testis to confirm cross-reactivity to avian tissue, and we observed positive signal for each antibody (Fig. S1). Specifically, anti-DAZL produced cytoplasmic, punctate signal in germ cells (Fig. S1A and B)^12^. Anti-DDX4 labeled spermatogonia and round spermatids (Fig. S1C and D) as expected based on chicken^13^. For SOX9, nuclear signal was observed in presumptive Sertoli cells aligned along the basal lamina (Fig. S1E, F, and G) also consistent with the pattern previously observed in chicken^14^.

**Fig. 5:**
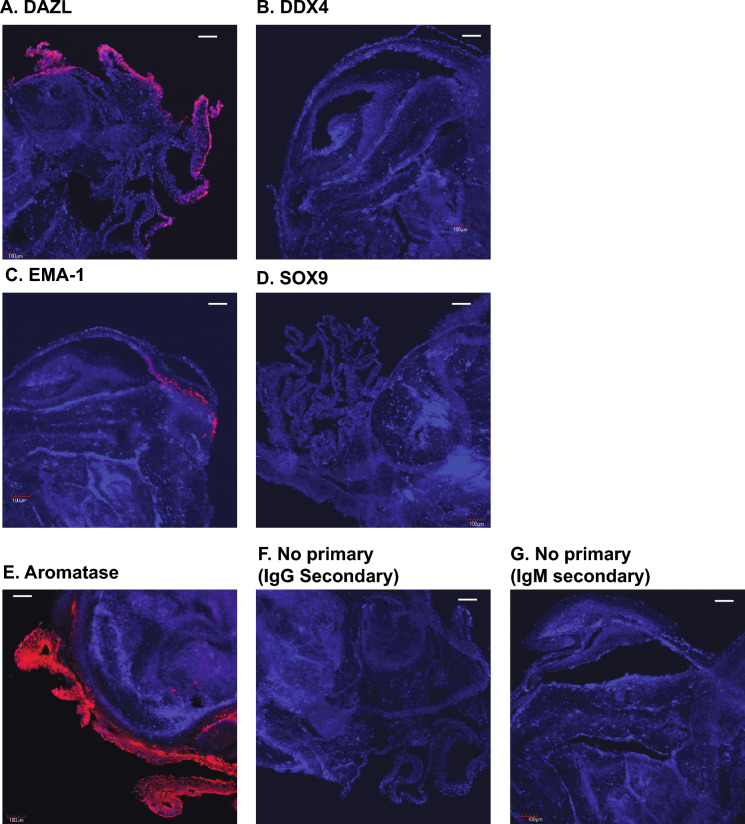
Immunofluorescence (red signal) in organoid E2 (female) at 147 days of culture. DAPI (blue) counterstains DNA in cell nuclei. (**A**) anti-DAZL immunofluorescence (red), 10x magnification. (**B**) anti-DDX4 immunofluorescence (red), 10x magnification. (**C**) anti-EMA-1 immunofluorescence (red), 10x magnification. (**D**) anti-SOX9 immunofluorescence (red), 10x magnification. (**E**) anti-Aromatase immunofluorescence (red), 10x magnification. (**F**) No-primary control with IgG secondary (control for antibodies targeting DAZL, DDX4, SOX9, and Aromatase). (**G**) No-primary control with IgM secondary (control for EMA-1 antibodies). All scale bars are 100 µm.

**Fig. 6:**
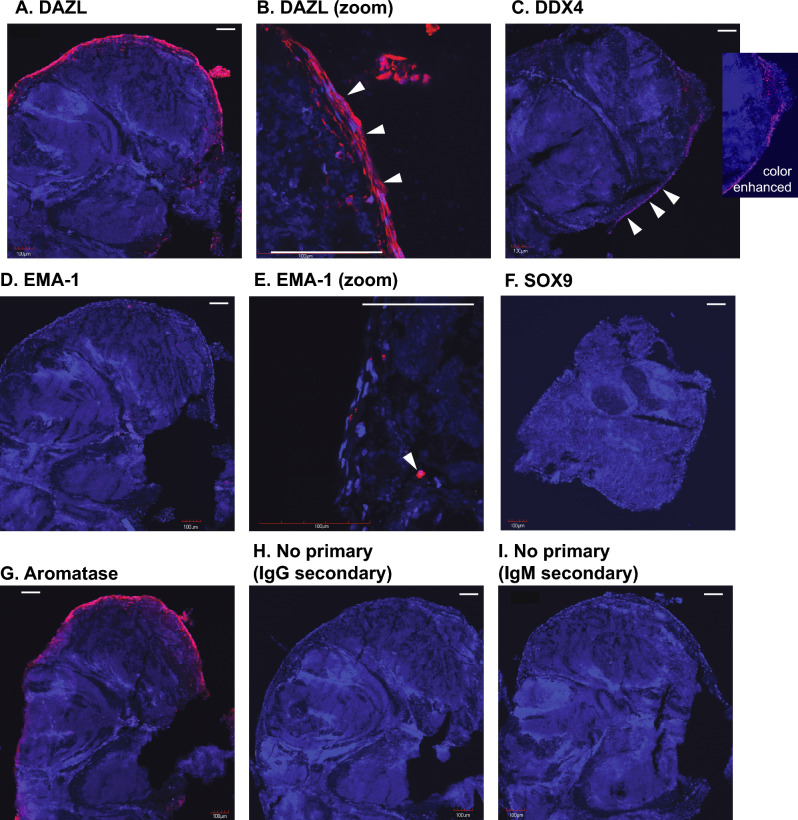
Immunofluorescence (red signal) in organoid AA (female) at 82 days of culture. DAPI (blue) counterstains DNA in cell nuclei. (**A**) anti-DAZL immunofluorescence (red), 10x magnification. (**B**) anti-DAZL immunofluorescence (red), 60x magnification. Arrowheads indicate the epithelial surface where DAZL signal is strong. (**C**) anti-DDX4 immunofluorescence (red), 10x magnification. Arrowheads indicate DDX4-positive epithelial surface. Inset: the intensity of color was increased for both red and blue channels to enhance visibility of the DDX4 signal. (**D**) anti-EMA-1 immunofluorescence (red), 10x magnification. (**E**) anti-EMA-1 immunofluorescence (red), 60x magnification. Arrowhead indicates positive cell. (**F**) anti-SOX9 immunofluorescence (red), 10x magnification. (**G**) anti-Aromatase immunofluorescence (red), 10x magnification. (**H**) No-primary control with IgG secondary (control for antibodies targeting DAZL, DDX4, SOX9, and Aromatase). (**I**) No-primary control with IgM secondary (control for EMA-1 antibodies). All scale bars are 100 µm.

**Fig. 7:**
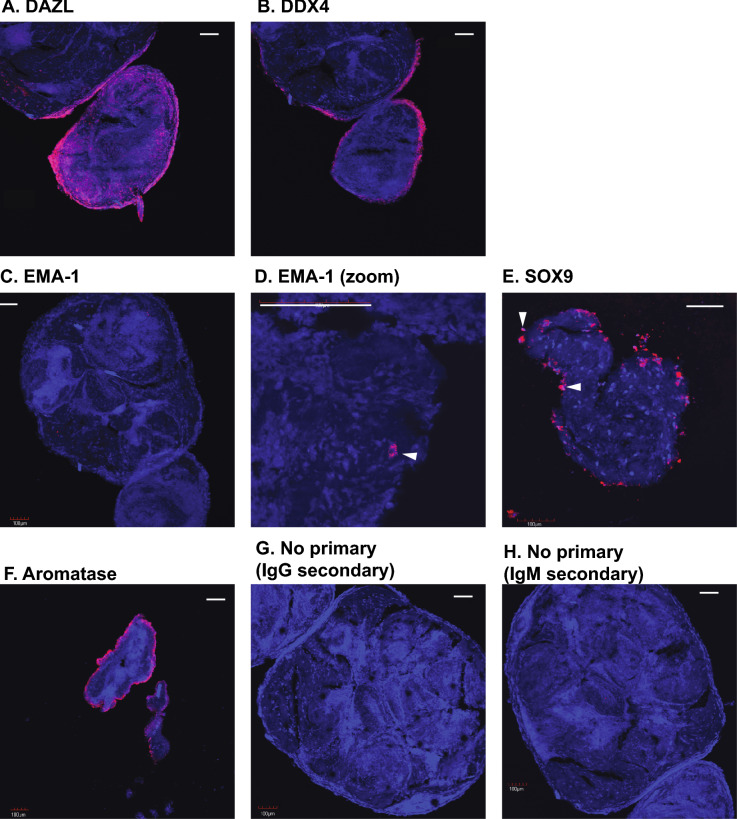
Immunofluorescence (red signal) in organoid D1 (male) at 64 days of culture. DAPI (blue) counterstains DNA in cell nuclei. (**A**) anti-DAZL immunofluorescence, 10x magnification. (**B**) anti-DDX4 immunofluorescence, 10x magnification. (**C**) anti-EMA-1 immunofluorescence, 10x magnification. (**D**) anti-EMA-1 immunofluorescence, 60x magnification. Arrowhead indicates positive cell. (**E**) anti-SOX9 immunofluorescence, 20x magnification. Arrowheads indicate positive cells. (**F**) anti-Aromatase immunofluorescence, 10x magnification. (**G**) No-primary control with IgG secondary (control for antibodies targeting DAZL, DDX4, SOX9, and Aromatase). (**H**) No-primary control with IgM secondary (control for EMA-1 antibodies). All scale bars are 100 µm.

**Fig. 8:**
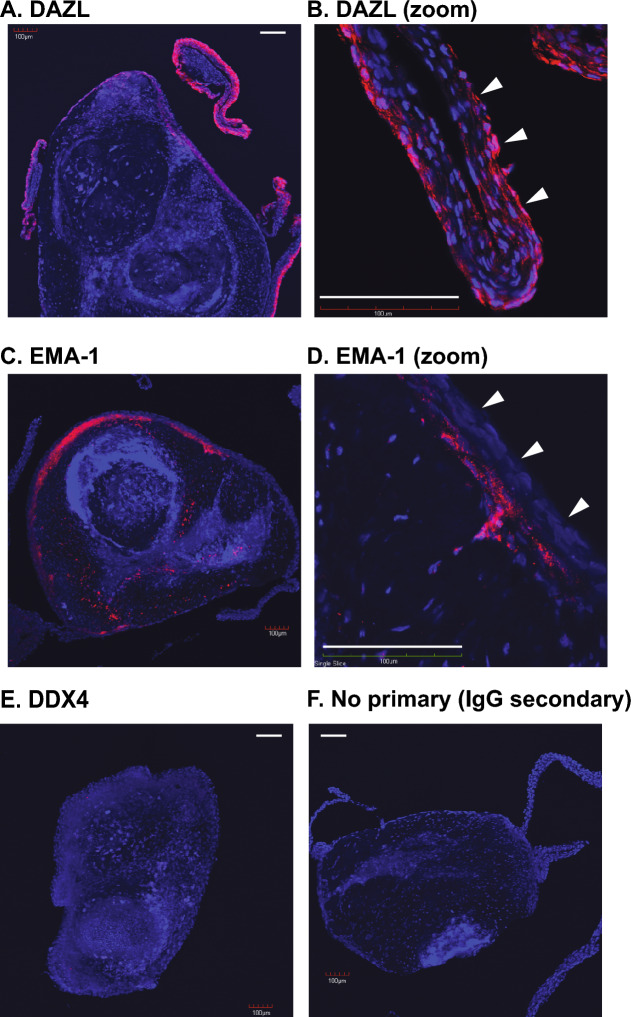
Immunofluorescence (red signal) in organoid J (male) at 120 days of culture. DAPI (blue) counterstains DNA in cell nuclei. (**A**) anti-DAZL immunofluorescence (red), 10x magnification. (**B**) anti-DAZL immunofluorescence (red), 60x magnification. Arrowheads indicate the epithelial surface where DAZL signal is strong. (**C**) anti-EMA-1 immunofluorescence (red), 10x magnification. (**D**) anti-EMA-1 immunofluorescence (red), 60x magnification. Arrowheads indicate epithelial surface with EMA-1 marking a distinct layer of cells relative to DAZL (panel B). (**E**) anti-DDX4 immunofluorescence (red), 10x magnification. (**F**) No-primary control with IgG secondary.

We therefore investigated four organoids by immunofluorescence: two male (organoids J and D1) and two female (organoids AA and E2). We observed significant autofluorescence in fixed organoid tissue at green (490nm / 595nm) and orange (590 nm / 618nm) wavelengths but this was eliminated at longer wavelengths (data not shown). We therefore utilized far-red AlexaFluor 647 (650nm / 671 nm) for visualization of all proteins, along with appropriate no-primary antibody controls (Fig. 5F,G, 6H,I, 7G,H and 8F).

For a summary of immunofluorescence results see Table 1. The most striking and consistent observation was a DAZL-positive outer layer, reminiscent of an epithelial layer surroundeding the organoids (Fig. 5A, 6A, 6B, 7A, 8A,B, Table 1). Two organoids were DDX4-positive, one male and one female (Fig. 6C and 7B, Table 1) and two were DDX4-negative, also one male and one female (Figs. 5B, 8E, Table 1). We note that the DDX4 signal was robust in male organoid D1 (Fig. 7B), and much lower in female organoid AA (Fig. 6C and inset) which we speculate may be due to the longer culture time of AA relative to D1 (Table 1). EMA-1, the PGC marker^15,16^, was generally found either in isolated scattered internal positions (Figs. 6D,E and 7C, & D) or, in a clusters just under the epithelial layer but near the surface (Fig. 5C, 8C, & D). Together these results reinforce the presence of both PGCs and later-stage germline cells around and within both male and female organoids.

The somatic gonadal genes SOX-9 and Aromatase present a valuable counterpoint to the germ-cell genes discussed above. (Unfortunately we were limited in material for organoid J, one of the male organoids, so it was not tested for these proteins (Table 1)). SOX-9 is a male-specific germline transcription factor critical for male sex determination^17,18^ and the male organoid tested (D1) exhibited SOX-9 positive cells (Fig. 7E). The female organoids were SOX-9 negative as expected (Table 1, Figs. 5D & 6F). Aromatase was detected in the epithelial-like layer in all organoids tested (Figs. 5E, 6G, 7F), consistent with its expression pattern in gonadal tissue of both sexes^19^ and confirmed in our testis sample (Fig. S1E).

**Fig. 9:**
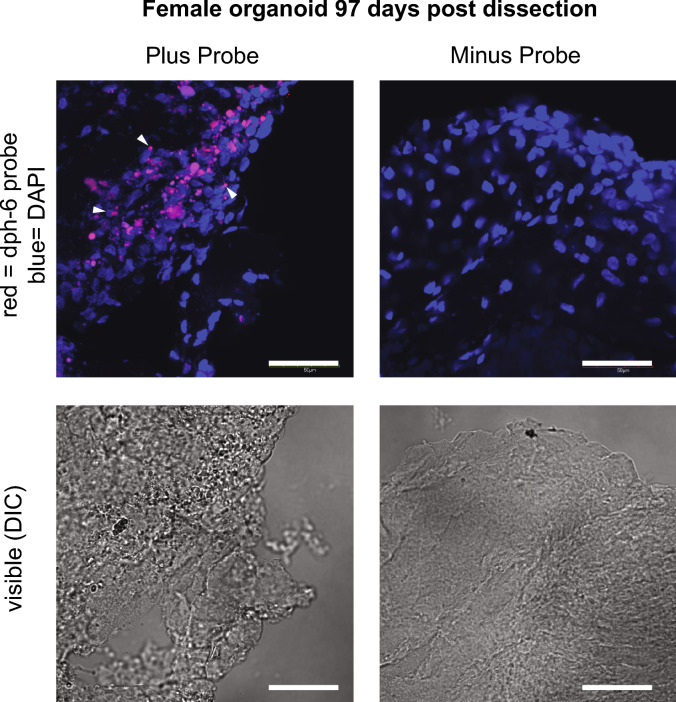
Fluorescence *in situ* DNA hybridization of the *dph-6* gene from the Germline-Restricted Chromosome (GRC). Organoid T, is female, at 97 days of culture. Probe was from^20^. White arrows highlight a few dph-6-positive Primordial Germ Cells which are present inside the organoid even after 97 days of culture. Scale bar, 50  µm.

For one organoid (organoid T), we tested for the presence of PGCs using Fluorescence In-Situ Hybridization (FISH) targeting the *dph-6* gene from the Germline-Restricted Chromosome (GRC) using methods previously demonstrated^20^. The GRC gene *dph-6* occurs hundreds of times, making it an excellent marker of this chromosome^20–22^. Multiple *dph-6* positive PGCs were present inside the organoid even after 97 days of culture, clustered in close proximity (Fig. 9).

**Fig. 10:**
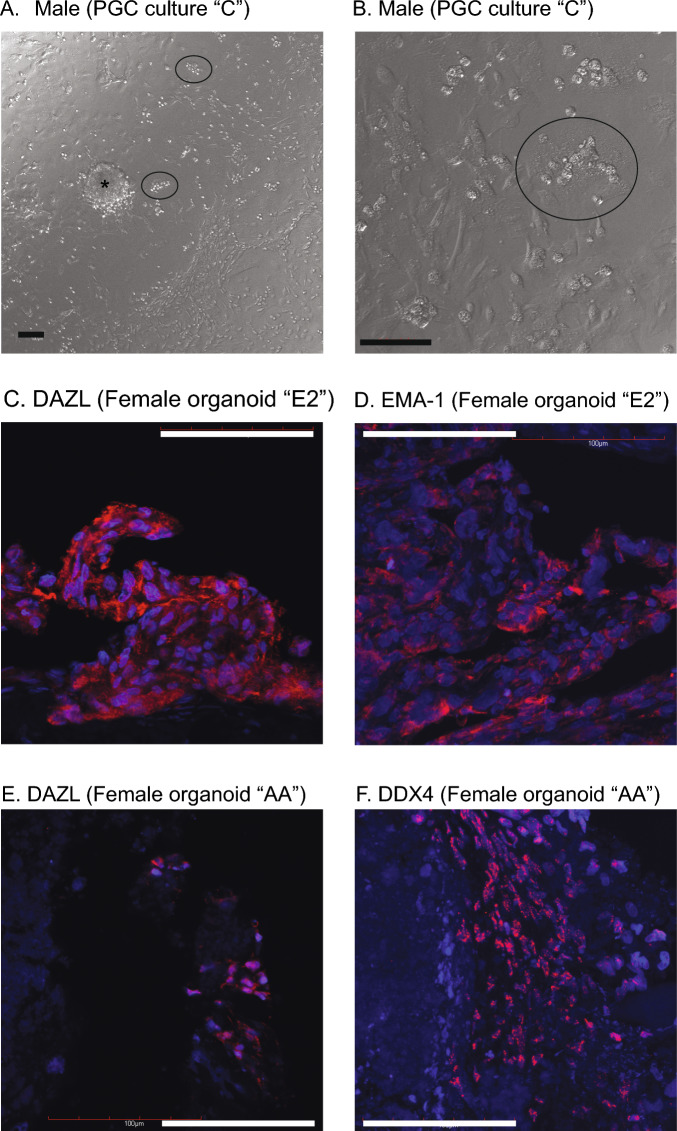
Visualization of PGC clusters in culture and in organoids. (**A**) DIC visualization of male PGC culture where an organoid (marked with asterisk) is forming along with several PGC clusters (circled). (**B**) DIC visualization of PGC cluster at higher magnification with a cluster circled. (**C**) anti-DAZL immunofluorescence (red) in female organoid E2. (**D**) anti-EMA-1 immunofluorescence (red) in female organoid E2. (**E**) anti-DAZL immunofluorescence (red) in female organoid AA. (**F**) anti-DDX4 immunofluorescence (red) in female organoid AA. For all panels nuclear DNA is labeled with DAPI.* Note* all scalebars = 100 µm.

**Table 1:** Detection of germline genes in organoids. Days indicates days in culture, from dissection to fixation for sectioning. dph-6 was detected by Fluorescence *In-situ* Hybridization (FISH). The remaining genes were detected by immunofluorescence. nd = not done.

Sex	Organoid	Days	DAZL	DDX4	EMA-1	SOX-9	Aromatase	*dph-6* (GRC)	Figures
Male	J	120	+	−	+	nd	nd	nd	Figure 8
Male	D1	64	+	+	+	+	+	nd	Figure 7
Female	AA	82	+	+	+	-	+	nd	Figure 6
Female	E2	147	+	−	+	-	+	nd	Figure 5
Female	T	97	nd	nd	nd	nd	nd	+	Figure 9

We noted several additional examples of highly clustered cells within the PGC cultures (Fig. 10A & B) and also clustered DDX4 and DAZL-positive cells within organoids (Fig. 10 C–F). Because of the close proximity of many of these cells (Fig. 10F), they are reminiscent of the DDX4-positive germ-cell nests observed in mammals which are linked by incomplete cytokinesis^23^. Future work is needed to examine potential cytoplasmic bridges linking these cells.

**Fig. 11:**
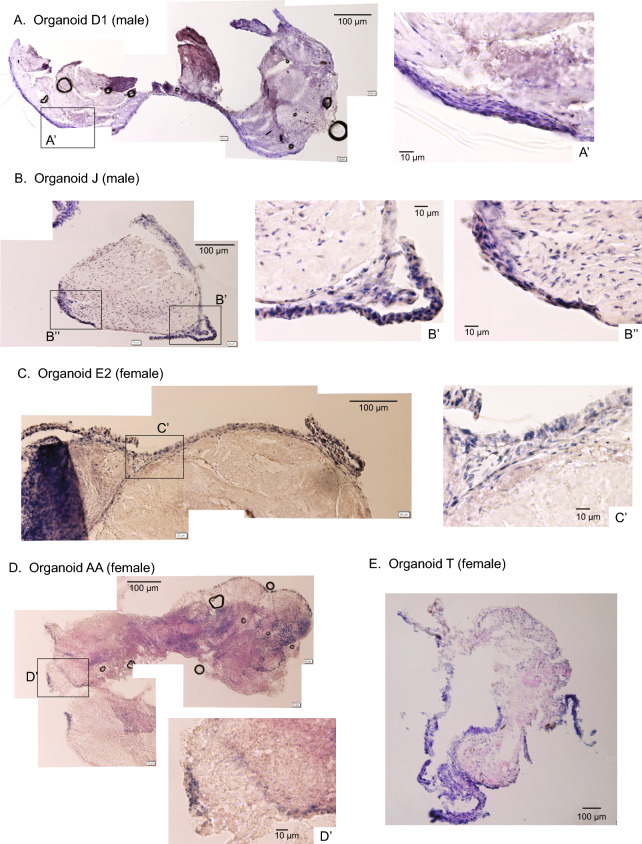
H&E staining of organoids. (**A**) Organoid D1 (male). (**B**) Organoid J (male). (**C**) Organoid E2 (female). (**D**) Organoid AA (female). (**E**) Organoid T (female). For all figure panels, insets indicate regions expanded at higher magnification and indicated as prime (′) or double prime (″).

We performed Hematoxylin and Eosin (H&E) staining on all five organoids from Table 1 (Fig. 11).The epithelial structures stained strongly while organoid interiors were generally less densely marked, containing internal chambers (Fig. 11). The darkly staining epithelial layer is consistent with a dense population of germline cells as confirmed by DAZL, DDX4, and EMA-1 immunofluorescence (Figs. 5, 6, 7, 8 and Table 1).

**Fig. 12:**
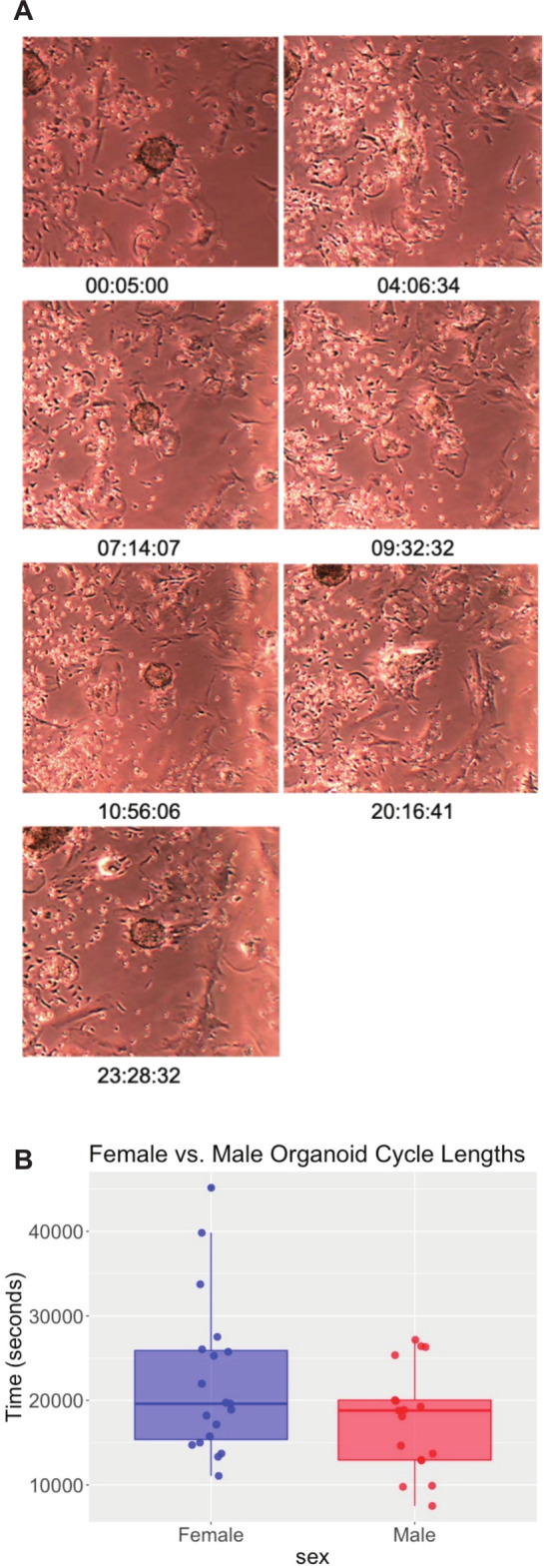
(**A**) Depiction of the opening and closing of an organoid multiple times over 23 hours in a single video. (**B**) Distribution of organoid cycle lengths (n=36). A total n=7 of the organoids are female (19 cycles) and n=8 are male (17 cycles). There is no significant statistical difference in distribution between male and female cycle lengths (*p* = 0.0923, Welch’s two-tailed T-test).

The ability to observe the behavior of living PGCs and organoids in culture enabled us to make observations that would be very challenging *in vivo*. For example, we observed a dynamic behavior in the organoids characterized by cycles of “opening” and “closing” which correspond to a process of cell adhesion and release. Figure 12A depicts this phenomenon (3 complete cycles) over a 23-hour time frame. We measured this opening and closing behavior for 15 total organoids (n=7 female and n=8 male), collectively yielding 36 cycles (Fig. 12B). Statistical analysis revealed no significant difference between male and female cycle lengths (*p*=0.0923, Welch’s two-tailed T-test), with cycles lasting approximately 5.5 h for both (Fig. 12B). While the potential functional significance of this behavior remains unknown, it has been shown that mammalian PGC cysts are comprised of PGCs from different mitotic ancestors cells, and are not mitotic sisters^23^. We hypothesize that dynamic organoid opening and closing may, *in vivo*, facilitate this reorganization of PGCs within nascent nests. We speculate that the opening process may contribute to the breaking apart of cytoplasmic bridges linking PGCs, allowing opportunity to form new combinations of PGCs from different cell lineages to be sequestered within organoids (at the closing phase) where they might link together in novel combinations as previously observed^23^. Further research is needed to clarify the biological role of this cycling of organoids.

Primordial germ cells are known to migrate long distances within a developing embryo to locate the gonadal ridge^24,25^. We therefore monitored migration within early-stage organoid cultures (male videos were 9 and 10 days since dissection while the female videos were of cultures 11, 13, and 19 days from dissection). We evaluated n = 3 male and n = 4 female organoids from these videos using a custom in house PGC-tracking program (available at: https://github.com/brachtlab/PGC-Organoid-Tracking) to see whether the cells are actively recruited to the structures they later inhabit. We found that motion relative to organoid was relatively random, and that if anything the motion was predominantly away from organoids (Fig. S2D). We conclude therefore that PGC attraction is not a feature of the cells in these cultures. This makes biological sense because PGC migration through the bloodstream occurs quite early in development, at HH stages 16-18, and PGCs settle into the gonadal ridge by HH 26-28^26^. Given that our cultures were from embryos at later developmental stages (HH stages 32-40) the PGCs may not be highly migratory in our culture, instead having entered a replicative nest- and epithelium- building phase.

## Discussion

Organoids offer important research insights into complex biological processes, and have been characterized from many somatic tissues including brain^27,28^, gut^29^, prostate^30^, liver^31^ and tumors^32,33^ among others. Most current knowledge of germline organoids comes from mouse and human models, while avian germline organoids have not been described to date. However, mammalian germline organoids provide important insights into the mammalian germline development and function. For example, two mouse papers have highlighted their value in toxicological studies^34^ and in restoring spermatogenesis^35^. Another paper highlights the ability of 3D-printed scaffolds to stabilize and long-term culture testicular mouse organoids^36^ while another mouse study shows that ovarian organoids can be produced from spermatogonial stem cells, in a surprising transdifferentiation^37^. Human testicular organoids^38–41^ and ovarian organoids^42–44^ mark significant progress in understanding the biology of human fertility.

In the current study we show that organoids spontaneously develop in germline cultures from both sexes of zebra finch (*Taeniopygia guttata*). This is surprising because in mammalian systems, formation of organoids nearly always requires a 3D substrate such as Matrigel^36,38,41,44^. In contrast, we show that for zebra finch germline organoids 3D culture and special substrates like Matrigel^35^ are not required and we did not employ estrogen receptor antagonists (such as ICI 182780), which have been utilized by some researchers^45^.

Supporting the more cell-autonomous behavior of the avian model, in other arenas mammalian and avian developmental mechanisms are quite divergent. For example, avian sex determination occurs primarily through cell-autonomous genetic mechanisms^46,47^, an evolutionary difference from mammals where hormones play a more central role^46^. Similarly, in some animals including birds, PGC fate is established by inheritance of maternally deposited cytoplasmic factors (preformation), but in others, including mammals, PGC fate is established through signaling from surrounding tissue (induction)^3,48^. Thus both for sex determination and PGC cell fate establishment, secreted signals and hormones are more central in mammals while genetic and inherited programming play central roles for birds.

This is important because in Fetal Bovine Serum (FBS)-supplemented cell culture, the level of hormones (like estradiol) can be highly variable and not well controlled^49,50^. Therefore, establishing an organoid model system that is less hormonally-driven would be advantageous. While removing steroid hormones by charcoal-stripping the FBS can be performed, this method has negative side effects of removing other lipids, proteins, and supportive vitamins^51^. Therefore, we did not use charcoal stripped FBS; we also used phenol red-containing media, and phenol red has also been shown to be a mild estrogenic agonist^52^. We hypothesize that avian germline development, consistent with bird sex determination^53,54^ and PGC specification^48^, may be more cell-autonomously determined than hormonally regulated. This would increase the robustness and reproducibility of organoid formation, consistent with our observations. A lower hormone dependency would position avian germline organoids as particularly valuable models for the study of vertebrate reproductive biology.

The study of vertebrate germline development presents significant challenges. This is because of both the length of time during which it occurs (from early embryonic stages to full sexual maturity of the adult) and also the intricate signaling interactions between cells and tissues. The establishment of PGCs occurs very early in embryonic development, and they arise in non-gonadal tissues^3,48^. In birds and reptiles the migration of PGCs is spectacular, as they circulate through the vasculature prior to homing in on their gonadal niches, while in mammals the PGCs must migrate long distances from extraembryonic epiblast where they originate, along the hindgut to the gonadal ridge^55,56^. Following localization in the gonadal ridge, PGCs undergo mitotic proliferation forming “nest” or “cyst” structures wherein incomplete cytokinesis renders daughter cells physically linked into PGC clusters, which proceed to enter meiosis^23^. The nests (PGC clusters) then break down; in females a single member of each cluster is incorporated into a primordial follicle, surrounded by somatic pregranulosa cells^57,58^. In males the cluster of PGCs become spermatogonia and ultimately spermatocytes^23,59,60^.

The organoids documented in this study are produced by germline tissue extracted from embryos after PGC migration into the genital ridge, when nest formation is underway *in vivo*. Consistent with this, we identified clusters of DDX4 and DAZL-positive cells (Fig. 10C & F) and the migration of PGCs *in vitro* was minimal with most migration observed being distant cells moving away from the organoid (Fig. S2). It remains to be seen whether this indicates a pro-mitotic role for the organoid as our tracking software did not explicitly monitor cellular divisions.

Our immunofluorescence experiments demonstrate that the organoids we describe are germline-marker positive, with strong signal in epithelial structures (Figs. 5, 6, 7 and 8) that also stain strongly with hematoxylin (Fig. 11). These epithelial structures are suggestive of the germinal epithelium, a conserved feature documented in developing embryonic gonads of birds, mammals, fish, and amphibians^61^. The defining feature of the germinal epithelium is that it is truly epithelial, covering the outer surface of the developing gonad; it forms in both sexes and contains the developing germ cells and the associated germline-specific somatic support cells (such as Sertoli or granulosa cells)^61^. Organoids displaying epithelial structures containing cells positive for DAZL, DDX4, Aromatase, and SOX-9 support the notion that these organoids may, in fact, exhibit germinal epithelium^61^.

The difference in expression between EMA-1, DAZL, and DDX4 is striking. In avian models, EMA-1 was shown to mark very early (migratory) PGCs and to be reduced once colonization of the gonad occurs^62^. Meanwhile DAZL is expressed throughout development from gastrulation to adult in germline lineages^10^. We observed the loss of EMA-1 from epithelia which nevertheless remain DAZL- and DDX4- positive (Organoids AA & D1, Figs. 6 & 7) which suggests that the germ cells have lost their early migratory PGC identities, but robust later-stage germ-cell populations remain, consistent with the timing of embryonic tissue harvest. In a zebra finch single-cell sequencing study two gonadal populations of PGCs were identified: zGC1 (DDX4-high) and zGC2 (DAZL-high)^63^. The authors conclude that zGC1 encodes a stem-cell population while zGC2 constitute a population of proliferative gonadal progenitor cells, and the DDX4-high cells transition over time into DAZL-high populations^63^. These data are consistent with the germline organoids we characterize here being largely at zGC2 stages characteristic of later (but still premeiotic) gonadal development. This is consistent with the fact that the two organoids (one male, one female) expressing DDX4 were also the youngest (Table 1), and between them, the younger one had much stronger DDX4 expression (compare Fig. 7B with Fig. 6C). Therefore, the differences in EMA-1, DAZL, and DDX4 expression are explicable based upon known expression timing differences *in vivo*.

While the relationship between nest formation and germinal epithelial formation is not well worked out, in teleost fishes nest formation precedes germinal epithelial formation^64,65^. This would support the order of events we see in organoid development in culture: first, PGC clusters can be identified (Figs. 1A,D and 10A,B) and then the organoids with their putative germinal epithelial structures form (Figs. 5, 6, 7 and 8). We note that adult gonadal structures do not form in our culture suggesting that some developmental cue from the embryo is needed to generate mature seminiferous tubules or ovarian follicles, and future work is needed to decode this mechanism.

## Methods

### Cell culture

Primordial Germ Cell Cultures were changed within a sterile hood every 4 days. A 1.5 mL microcentrifuge tube was labeled for each respective culture within the 24-well culture plate (each well holds 1ml). The entire content of each well was carefully transferred to its corresponding 1.5ml microcentrifuge tube. Immediately after removing the contents of each well, 500ul of fresh PGC media was gently added back to the well (pipetting along the side wall), ensuring the adherent cells did not dry out. Each microcentrifuge tube was then centrifuged at 200g for 3 minutes at room temperature and the supernatant was aspirated, leaving 100ul behind. Gently, a fresh 400ul of fresh PGC media was added to each tube with gentle mixing by pipetting. The media in each microcentrifuge tube was pipetted up and down carefully before being placed back into their respective well (reconstituting the 1ml starting amount).

### Egg collection and dissection

All protocols were approved by American University IACUC. Eggs were collected from their respective mating cage located in American University’s onsite aviary every day of the workweek. These eggs were placed into a chicken incubator set at 37° C and rotates the collected eggs every two hours. For humidity the tray is maintinaed with deionized water. Seven days after egg collection dissection is performed to retrieve embryonic gonad tissue, at which point the embryo should be developed between Hamburger-Hamilton stage 32 to 40 (HH32-40). (We find the eggs have some delay in development which induces some variability into the staging). Upon breaking open the eggs, embryos were visually staged based upon a previously published study^66^. The dissection method to retrieve gonad tissue has been adapted from protocol established by The Laboratory of Neurogenetics of Language based at The Rockefeller University and previously published^10^.

The gonads of the embryos (both left and right, if possible) were collected as described^10^, with those from individual birds kept separate, into 500$\upmu$L of 0.05% trypsin solution, incubated at 37 °C for 5 minutes. The tissue was dissociated manually by up-down pipetting for 15–20 times using a 200$\upmu$L pipette. Following dissociation the trypsin was inactivated with the addition of 500$\upmu$L PGC media (as described in^10^), the samples were centrifuged at 200 rcf for 3 minutes, and all but 100$\upmu$L of supernatant was removed. To prepare the samples for culturing, the cells were resuspended in 900$\upmu$L of PGC media and split between two wells on a 24-well cell culture plate (Greiner Bio-One Cat # 662160) that already contained 500$\upmu$L of PGC media. This resulted in each embryo producing two culture wells with 1mL of solution each. The remaining embryonic tissue was stored in -80 °C in order to be sexed later on in the experimental process.

### Sex detection PCR

DNA for PCR was obtained from the frozen embryos. We found that the easiest way to obtain the DNA was simply defrosting the embryo, pipetting 10-20 $\upmu$L of PBS from around the embryo, which was re-frozen. The 10-20 $\upmu$L of PBS was purified on Zymo RNA Clean and Concentrator-5 (catalog number R1015) to remove PCR inhibitors. Elution was into 10$\upmu$L of water and the PCR was set up with 2$\upmu$L of this purified material. The sex-determination primers target CHD1. Primers were:

CHD1F: 5’-TATCGTCAGTTTCCTTTTCAGGT-3’

CHD1R: 5’-CCTTTTATTGATCCATCAAGCCT-3’

Amplification was carried out as shown in Table 1 using touch-down PCR:

**Table 2:** Touch-down PCR amplification protocol.

Step	Condition
1	94 °C, 4 min
2	94 °C, 1 min
3	57 °C, 1 min (decrease 1 °C per cycle)
4	72 °C, 1 min
5	Repeat steps 2–4, 8 times
6	94 °C, 1 min
7	48 °C, 1 min
8	72 °C, 1 min
9	Repeat steps 6–8, 29 times
10	72 °C, 5 min
11	12 °C, infinite hold

### Organoid video analysis and cycle length determination

To visualize organoid development and behavior in real time, we placed a Motic AE2000 compound microscope inside the CO2 incubator, running the cables through the ventilation opening in the back to connect with an external MAC Pro computer. A Moticam 5+ 5 megapixel digital camera attached to the microscope was used to capture video using Motic Images Plus 3.0 software. Recordings ran for 16-24 hours. To track open/closing cycles for a given organoid, the timestamps at which an organoid appeared to “open” or “closed” were collected. In this study, an open organoid’s outer boundary is difficult to define and/or an irregular stretched shape while a closed organoid contains a solid outer boundary and a mostly circular shape. The time period between two subsequent “open” orientations in an organoid will be referred to in the remainder of this paper as an organoid’s cycle length. For statistical comparison between male and female, a Welch’s two-tailed T-test was performed.

### Media

PGC media was made as previously described^10^ with the addition of a final filter sterilization step, using a 0.2µm filter (VWR/Avantor Cat #10040-436).

### Organoid size determination with Image J

The organioids were imaged on a calibrated Motic AE2000 compound microscope and photos were taken with a scale bar. These images were processed in ImageJ version 1.54h (http://imagej.org) as follows. *Calibration (only do this once)* open (”file”, then ”open”) a scale-bar-containing image, and draw a line over the scale bar matching its length (there is a line-drawing option on the toolbar when the software starts up). Then while the line is highlighted, click on ”analyze” and then ”set scale”. Enter 100 for the known length (if it is 100 µm) and micron as the unit. Make sure to click ”global” box so that other images (assuming they are at the same magnification) will use the same scale. Then select ”ok” to set the calibration. *Analysis (do this for each image)* To analyze organoid size, open the image you want (”file”, then ”open”). Select ”image”, then ”type” and 8-bit. This converts to greyscale. Then go to ”image”, ”adjust”, ”threshold”. This will bring up a menu and will automatically try to set a threshold (make sure ”dark background” is NOT selected). The goal here is for the organoid to be totally white on a dark background, you can adjust the two sliders to set an appropriate range. Once settings are correct click ”apply” and close. Then go to ”analyze” and ”analyze particles”. Make sure ”Display results” is selected. Also, the ”Show” menu should be set to”outlines”. Click ”ok” to run the analysis. Two things show up: a map with labeled outlines of particles, and a set of numbers showing sizes in square microns. Find the size for the organoid outline in the output results and record it in a spreadsheet. Repeat this process for each image.

The data from ImageJ was saved as comma-separated file, then imported as a data frame into R (4.4.3) in Rstudio (v. 2023.12) for plotting with the loess() function in ggplot2.

### Fixation and sectioning of organoids and testis tissue

Organoids or freshly-dissected testes were fixed in 4% paraformaldehyde in PBS overnight. Following fixation samples were washed 3x in PBS, then transferred to 10% sucrose in PBS overnight at 4 °C. The next day they were transferred to 20% sucrose in PBS, stored at 4 °C overnight. The next day they were transferred to 30% sucrose in PBS, stored at 4 °C overnight. The sucrose-embedded organoids or testes were sectioned on a CryoStar NX70 cryostat by embedding into O.C.T. media (Tissue-Tek Cat # 4583) and sectioning into 25 µm sections which were captured on superfrost slides (VWR Cat # 48311-703). Slides were stored at −20 °C until use.

### Fluorescence in-situ hybridization (FISH)

For GRC staining by FISH, the previously published protocol from^20^, as detailed in^67^ was used but with minor modifications. Upon removal of slides from −20 °C, they were warmed to 50 °C for 15 minutes to improve attachment of section to the slide. Subsequently, two washes of PBS were used to remove residual O.C.T. media from the slide sections (about 30 seconds each wash) prior to hybridization. The remaining protocol from^67^ on page 414 and following was followed as written, but the pepsin was increased to 2% in PBS, following^68^, and the pepsin digest increased, previously 2–5 min but brought to 15 min at 37 °C.

The FISH probe was constructed using an ARES AlexaFluor 647 DNA labeling kit (ThermoFisher Catalog # A21676), by nick translation. The DNA was constructed by PCR using dph-6 primers as described in^20^ and the manufacturer’s instructions were used in all steps for nick translation, except purification was performed using Zymo RNA Clean and Concentrator-5 (catalog number R1015), not by ethanol precipitation. Successful probe labeling was confirmed by a NanoVue Plus Spectrophotometer prior to use.

### Immunofluorescence

Buffers used:

- *Blocking & Permeabilization Buffer (BPB)* PBS 1x (Gibco Cat # 10010031) 10% BSA 0.3% Triton X-100
- *Antibody Dilution Buffer (ADB)* PBS 1x (Gibco Cat # 10010031) 1% BSA 0.3% Triton X-100

(Note 1, Gibco Cat # 10010031 PBS is 1.06mM potassium phosphate monobasic (KH2PO4), 155mM NaCl, and 3mM sodium phosphate dibasic (Na2HPO4-7H2O)).

(Note 2, PBST is PBS supplemented with 0.1% Triton X-100.)

After removal from the −20 °C storage, slides with organoid or testis sections were warmed to room temperature for five minutes. The slides were warmed on a heat block for 15 minutes at 50 °C to strengthen adherence to the superfrost slide (VWR Cat # 48311-703). The sections were then washed twice in PBST for 30 seconds per wash to remove residual OCT media. They were incubated in a moist chamber (parafilm-sealed 100mM plastic petri dish with dampened kimwipe) in Blocking and Permeabilization Buffer (BPB) for one hour at 37 °C. The BPB was removed and then primary antibodies, diluted in Antibody Dilution Buffer (ADB) to the concentrations shown in Table 2 below, were added. Samples were incubated at 4 °C overnight in the moist chamber. The following day, the sections were washed 3 times for 5 minutes in PBST, then incubated in secondary antibodies for 1 hr at room temperature. The samples were then washed 3 more times for 5 min per wash in PBST, excess liquid gently blotted, and mounted with glyerol mounting media with DAPI (Electron Microscopy Science, Cat # 17989-60). The slides were covered with a coverslip and sealed with clear nail polish before imaging on an Olympus FV1200 scanning confocal microscope under 10x and 60x magnification. Because of significant autofluorescence we observed at other wavelengths, all imaging was performed with the AlexaFluor 647 nm secondary.

**Table 3:** Antibodies used in the study.

Gene	Company	Catalog #	Immunization host	Immunogen species	Percent identity of immunogen to Zebra Finch	Dilution used for immunofluorescence
DDX4 (VASA)	Abcam	ab209710	Rabbit	Zebrafish	67%	1:100
DazL	Abcam	ab34139	Rabbit	Mouse	60%	1:50
Sox9	Avantor	76761-370	Rabbit	Human	97%	1:100
EMA-1	DSHB	AB_531885	Mouse	Mouse	n.d.	1:4
Aromatase (AZAC)	–	–	Rabbit	Finch	100%	1:500
Anti-mouse secondary 647 nm	Invitrogen	A-21238	Goat	Mouse	–	1:500
Anti-rabbit secondary 647 nm	Invitrogen	A32733	Goat	Rabbit	–	1:500

Aromatase antibody was provided by Colin Saldanha as a collaborative reagent.* AZAC* Anti-Zebra finch Aromatase C-terminal.* DSHB* Developmental Studies Hybridoma Bank.* n.d.* not done.

### H&E staining

After removal from the −20 °C storage, slides with organoid sections were warmed to room temperature for five minutes. The slides were warmed on a heat block for 15 minutes at 50 °C to strengthen adherence to the superfrost slide (VWR Cat # 48311-703). Section-bearing slides were immersed in the following, in 50ml Falcon tubes (Table 4):

**Table 4:** H & E staining protocol.

Step	Treatment	Duration
1	70% Ethanol	3 min
2	Deionized water	30 sec
3	Hematoxylin solution (Poly Scientific, Cat # 212A)	4 min
4	Deionized water	1 min
5	Acid Ethanol (30 mM HCl in 70% EtOH)	1 min
6	Scott’s Tap Water (166mM MgSO4 and 50mM NaOH in tap water)	1 min
7	Deionized water	2 min
8	Eosin Solution (Poly Scientific, Cat # 176)	45 sec
9	95% Ethanol	15 min
10	100% Ethanol	15 min

Air dry and mount with glycerol mounting media (Electron Microscopy Science, Cat # 17989-60), and seal with clear nail polish for imaging. Composite images were created using Panorama Stitcher Mini v. 1.11.2.

## Supplementary Information

Supplementary Information 1.

Supplementary Information 2.

## Data Availability

Data generated in this study is available by contacting the corresponding author, John Bracht (jbracht@american.edu).

## Code availability

All code, documentation, and related genomic data are available in GitHub page of the Bracht lab, “PGC-Organoid-Tracking” https://github.com/brachtlab/PGC-Organoid-Tracking
