# Supplementary material for: Germline organoids develop in vitro from embryonic Taeniopygia guttata (zebra finch) cultures
Source: Sci Rep. 2026 Apr 20;16:18401. doi: 10.1038/s41598-026-46600-z (PMC13265798; doi:10.1038/s41598-026-46600-z)
Supplement: Supplementary file 1 — Supplementary Information 1. [file 41598_2026_46600_MOESM1_ESM.pdf]

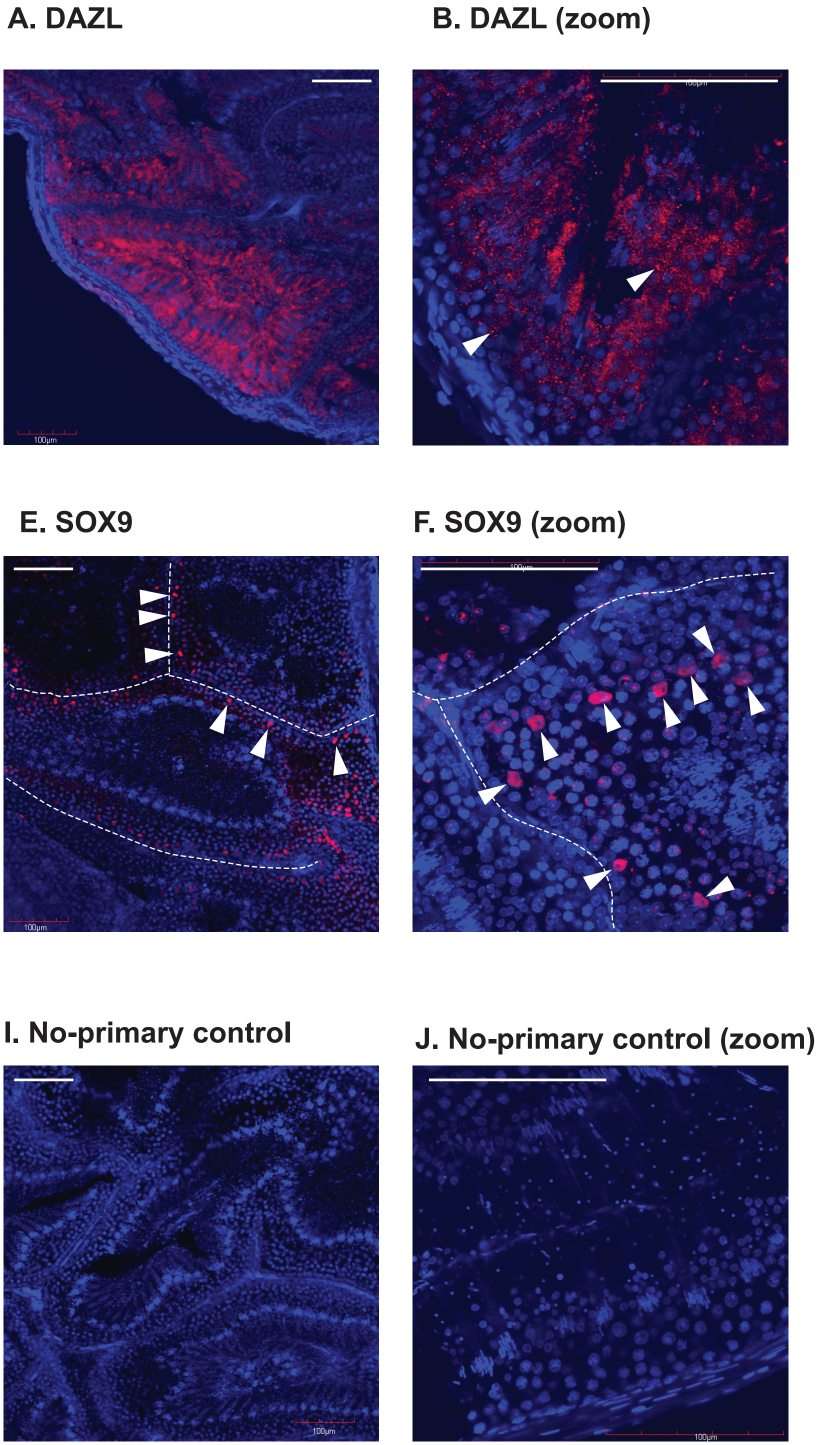

Figure S1: Testis immunofluorescence to confirm novel antibodies function in zebra finch tissue. **A**: anti-DAZL immunofluorescence (red). 20x magnification. **B**: anti-DAZL under 60x magnification. Arrows indicate cytoplasmic granules. **C**: anti-DDX4 (red) at 20x magnification. **D**: anti-DDX4 (red) at 60x magnification. Shown are DDX4-positive spermatogonia at basement membrane (arrowheads) and round spermatids (asterisks).**E:** anti-SOX9 immunofluorescence (red) marks presumptive Sertoli cells (arrowheads) adjacent to the basement membrane (dotted line). **F:** anti-SOX9 zoomed in to show nine presumptive Sertoli cells (arrowheads). **G:** DAPI only for the image from (F) to show the diffuse irregular nuclei where the anti-SOX9 signal localizes (arrowheads, same locations as F). **H**: anti-Aromatase (red), 20x magnification. **E**: No-primary control, 20x magnification. **J**: No-primary control, 60x magnification. For all panels, the scale bar = 100 μm.
