# Supplementary material for: Germline organoids develop in vitro from embryonic Taeniopygia guttata (zebra finch) cultures
Source: Sci Rep. 2026 Apr 20;16:18401. doi: 10.1038/s41598-026-46600-z (PMC13265798; doi:10.1038/s41598-026-46600-z)
Supplement: Supplementary file 2 — Supplementary Information 2. [file 41598_2026_46600_MOESM2_ESM.pdf]

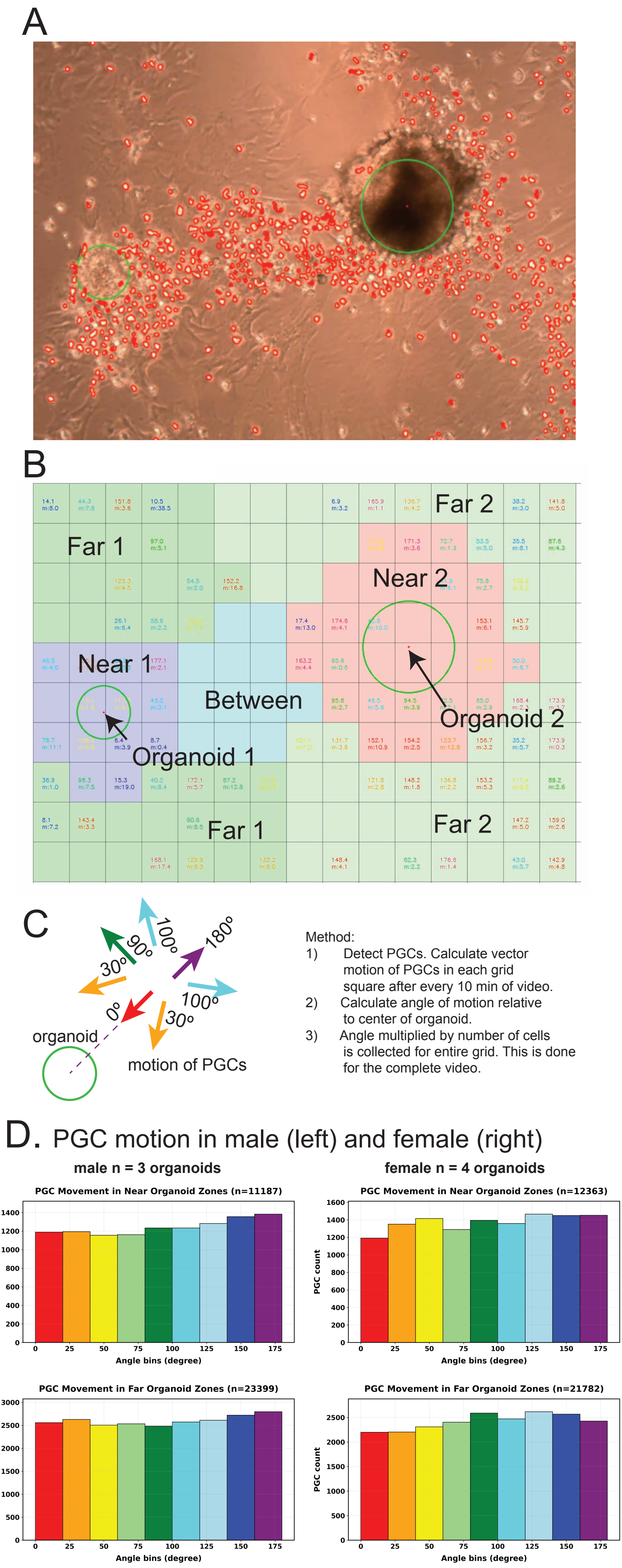

Figure S2: Custom software for cell tracking of PGC motion relative to organoid. **A**: Example of PGC identification in male 10-day old PGC culture. PGCs are outlined in red and organoids are green circles. (There are two organoids in this video). Videos were subsampled to 0.2 f.p.s, or one frame every 5 seconds. **B**: Grid structure used in analysis. Regions plotted as colors: Near organoid 1 (purple), Near organoid 2 (pink), Far organoid 1 (dark green) and Far organoid 2 (light green); between (light blue) was excluded from analysis. **C**: Schematic showing angle calculation, using organoid-centric radial reference frame. Quantification of cell motions was performed for each square every 120 frames (10 min), by calculating an average vector of the motion of all cells in the grid. This vector was compared to the angle relative to center of the organoid, with 0 degrees indicating motion directly toward, and 180 degrees directly away, from organoid. (Note, the angle folds back after 180 degrees to enable us to capture relative motion toward or away the organoid. Thus, what would be 200 degrees (180+20) is actually 160 degrees (180-20), 250 degrees (180+70) would be 110 degrees (180-70), and so on.). This angle is multiplied by the number of cells tracked and these values are collected for the entire grid. This is repeated every 120 frames for the entire video (23 hours) to generate the data for panel (D). **D**. Histogram of PGC motions in n = 3 organoids (male, left plots) and n = 4 organoids (female, right plots). The distribution of male "near" vs. "far" zones is statistically significant both by non-parametric and parametric test (p-value = 0.0094, Mann-Whitney U, and p-value = 0.0092, Welch's t-test) whereas the female distributions are undistinguishable from each other (p-value = 0.8327, Mann-Whitney U-test, p-value = 0.8019, Welch's t-test).
